# Channel–Spatial Fusion Attention for Wind Field Prediction in High-Rise Building Fire Scenarios

**DOI:** 10.3390/s26092666

**Published:** 2026-04-25

**Authors:** Sheng Zhang, Zhengyi Xu, Jianming Wei

**Affiliations:** 1 Shanghai Advanced Research Institute, Chinese Academy of Sciences, Shanghai 201210, China; zhangsheng12023@shanghaitech.edu.cn; 2 School of Information Science and Technology, ShanghaiTech University, Shanghai 201210, China

**Keywords:** wind field prediction, high-rise building fire, artificial neural network, attention mechanisms, multilayer perceptron

## Abstract

To improve the predictive accuracy of wind-field distributions during fires in high-rise buildings, this study targets the shortcomings of traditional prediction methods, including insufficient information fusion and dispersed feature representations under high-rise fire conditions. An efficient attention mechanism, termed Adaptive Channel and Multi-Scale Spatial Fusion Attention Mechanism (CSFAM), is proposed, which endows the model with enhanced adaptive focusing and multi-scale integration capabilities. CSFAM can account for environmental features across multiple dimensions to enable high-spatial-resolution wind-field reconstruction, thereby improving robustness and prediction accuracy in complex environments. To validate the effectiveness of CSFAM for predicting wind fields under high-rise-fire conditions, CFD-based scenario modeling was employed to generate a dataset of 1050 CFD-derived wind-field distributions across diverse inflow-wind and fire-source scenarios, partitioned into training, testing, and validation sets according to the fire-source size. When applying the CSFAM-enhanced multi-layer perceptron (MLP), the wind-field predictions achieved a mean squared error (MSE) of 0.0004, a mean absolute error (MAE) of 0.0141, and an R^2^ of 0.9766, outperforming state-of-the-art methods. The results demonstrate that CSFAM plays a significant role in markedly improving wind-speed prediction accuracy during high-rise-building fires, and enhances the model’s ability to identify and express vortex-like and other key aerodynamic features generated by the fire, thereby improving the capture of the complex nonlinear aerodynamic structures induced by fire.

## 1. Introduction

Although existing wind field prediction models for high-rise building fires have achieved certain progress, there remains considerable room for improvement in terms of prediction accuracy under complex fire environments and adaptability to diverse scenarios. Therefore, this paper proposes an attention mechanism that integrates adaptive channel attention with multi-scale spatial fusion, with the aim of significantly enhancing both the accuracy and robustness of wind field prediction.

### 1.1. The Importance of Wind Field Research

High-rise building fires are characterized by complex airflow dynamics, and the wind field distribution directly influences the effectiveness of rescue strategies [1,2]. Current prediction methods predominantly rely on numerical simulations or shallow statistical models [3], which exhibit limitations including limited real-time performance, high parameter sensitivity, and insufficient spatial feature representation [4]. Numerical simulations are unable to capture the instantaneous variations occurring within fire scenes [5], whereas shallow models fail to effectively exploit multi-scale and multi-regional interaction information, thereby limiting prediction accuracy [6]. These challenges substantially impede the effectiveness of fire emergency responses and hinder the timely and accurate provision of wind field information for decision-making [7,8].

### 1.2. Analysis of Existing Methods

Prediction of wind fields in high-rise building fires primarily depends on traditional computational fluid dynamics (CFD) and machine learning integrated CFD (ML-CFD) approaches [9]. Through years of development [10], CFD has revealed nonlinear dynamics and vortex structures in wind fields [11]. However, the high computational cost of CFD in large-scale complex environments remains a major bottleneck, restricting its real-time applicability [12].

Compared to traditional CFD, machine learning (ML) does not require an explicit physical foundation and can extract system behaviors directly from data [13], enabling near-instantaneous prediction of fluid dynamic states [14]. However, under fire-induced disturbances including varying coefficients, local heat sources, smoke concentration, and abrupt variations in local wind direction and velocity [15], a single ML model struggles to comprehensively capture the wind field’s spatial heterogeneity and temporal dynamics [16]. In particular, for multi-scale, nonlinear, transient [17], and disturbance-induced variations in wind field structure [18], traditional ML models frequently suffer from an “over-smoothing” effect, which weakens key regional features and degrades prediction accuracy and robustness [19].

Current ML-CFD methods predominantly employ global features or averaging, which simplifies model complexity but diminishes sensitivity to localized abrupt changes and high-frequency details near the fire source [20], thereby restricting response speed and prediction accuracy. See Table 1 for recent advances in CFD and ML-CFD [21]. To address this limitation, an Adaptive Channel and Multi-Scale Spatial Fusion Attention Mechanism is integrated to adaptively recalibrate channel weights [22], thereby enhancing focus on critical local features near the fire source, improving model responsiveness to spatial heterogeneity and nonlinear dynamics, effectively capturing high-frequency details, and significantly improving prediction accuracy in complex fire wind fields [23].

### 1.3. Contributions of This Paper

Existing high-rise building fire wind field prediction primarily relies on CFD simulations and conventional simple neural networks. A single CFD simulation is computationally intensive, often requiring hours to days. Conventional simple neural networks insufficiently account for input conditions, which readily results in inadequate fusion of multi-physical field features and loss of local details, thus reducing prediction accuracy.

Existing attention-based architectures are primarily designed for general image-processing or unstructured data tasks. They typically employ single-channel attention or spatial attention modules and seldom offer specialized adaptation to the strong thermo-fluid-building geometry coupling and highly nonlinear disturbances characteristic of high-rise fire wind fields. Therefore, the Adaptive Channel and Multi-Scale Spatial Fusion Attention Mechanism (CSFAM) proposed in this paper dynamically weights multi-physical input features through an adaptive channel fusion module and integrates a multi-scale spatial enhancement module to capture fire-source-induced local anomalies, thereby achieving deep fusion and adaptive enhancement of channel and spatial attention.

The innovation of this mechanism compared to existing attention mechanisms lies in the following aspects: (1) it is specifically designed for strongly coupled multi-physics scenarios, introducing adaptive channel weighting to enhance the interactive representation of key input conditions such as the fire source and inflow, rather than relying on generic static weighting. (2) it adopts a multi-scale spatial fusion strategy that prioritizes capturing local high-frequency disturbances while preserving global context, thereby avoiding the loss of high-frequency details or over-smoothing problems commonly observed in traditional attention mechanisms. (3) through a lightweight fusion structure, it significantly improves the representation capability and prediction robustness for nonlinear features in complex fire wind fields with only a limited increase in parameters, achieving higher accuracy while maintaining low computational cost.

### 1.4. Overview of the Paper Structure

Section 1 presents the research background and the current state of the art regarding wind fields in high-rise building fires, and explicitly states that the core innovation of this article lies in a cross-disciplinary breakthrough at the intersection of high-rise fires, wind fields, and neural networks. Section 2 explains the overall architecture of the Adaptive Channel and Multi-Scale Spatial Fusion Attention Mechanism (CSFAM). Section 3 describes the methodology and parameters for constructing the high-rise fire wind field dataset. Section 4 analyzes the composition of the dataset and presents the neural network’s predictive results. Section 5 provides a summary of the experiments.

## 2. Research Methodology

This section presents the architecture of the Adaptive Channel and Multi-Scale Spatial Fusion Attention Mechanism (CSFAM). CSFAM is composed of serially connected channel and spatial weighting modules that adaptively enhance crucial information and dynamically modulate the network’s receptive capacity. Additionally, this section examines the fusion of the multilayer perceptron (MLP) with CSFAM.

### 2.1. Neural Network Framework and Attention Mechanism

In high-rise fire wind fields, thermal energy released from the fire source induces complex dynamic responses characterized by nonlinearity, multi-scale features, and spatial heterogeneity, resulting in rapid variations in wind speed, wind direction, and vertical velocity.

The improved MLP, enhanced with an Adaptive Channel and Multi-Scale Spatial Fusion Attention Mechanism, adaptively regulates channel importance to dynamically integrate multi-channel features. This mechanism generates spatial and channel attention weights that emphasize physical regions influenced by fire-induced thermal disturbances-including local high wind speeds, intensified vertical velocity gradients, and areas of wind direction deviation-thus reinforcing critical feature representation. In complex terrain, interactions between terrain-induced wind direction deviations and fire thermal effects induce spatial heterogeneity. The attention mechanism dynamically identifies and propagates critical information, enhancing sensitivity to vertical motions, local wind patterns, and high wind speed zones.

By leveraging the attention mechanism, the improved MLP achieves deep integration of complex physical processes, capturing not only multi-scale and multi-channel features but also enhancing sensitivity to local thermal disturbances and vertical dynamics, thereby significantly improving the accuracy and physical consistency of high-rise fire wind field predictions.

### 2.2. Adaptive Channel and Multi-Scale Spatial Fusion Attention Mechanism

This section elaborates on the Adaptive Channel and Multi-Scale Spatial Fusion Attention Mechanism, covering its overall architecture, the algorithm for the channel weight module, and the algorithm for the Multi-Scale Spatial Enhancement Module.

#### 2.2.1. CSFAM Framework Introduction

To achieve a breakthrough in the interdisciplinary research direction of high-rise building fire + wind field + neural networks, the overall architecture of CSFAM is formed by sequentially connecting the adaptive channel fusion module and the multi-scale spatial enhancement module, specifically addressing from a macroscopic perspective the two core challenges in high-rise building fire wind field prediction: the coupling of multi-physical parameter inputs and the multi-scale heterogeneity and nonlinear characteristics of the wind field spatial distribution.

The adaptive channel fusion module is primarily responsible for addressing the weight distribution of multi-physical channels in high-rise fire wind field prediction. In high-rise fire scenarios, where the input features include multiple physical channels, this module can macroscopically and adaptively identify and reinforce the key channels that dominantly drive the overall wind field evolution, while suppressing the influence of secondary channels.

The multi-scale spatial enhancement module is primarily responsible for addressing the differentiation of spatial region importance in high-rise fire wind field prediction. This module captures the spatially critical regions of the fire-induced wind field across different scales, thereby effectively mitigating the smoothing-induced loss of spatial heterogeneity in traditional models and enhancing the prediction capability for local nonlinear details of the wind field.

**Figure 1:**
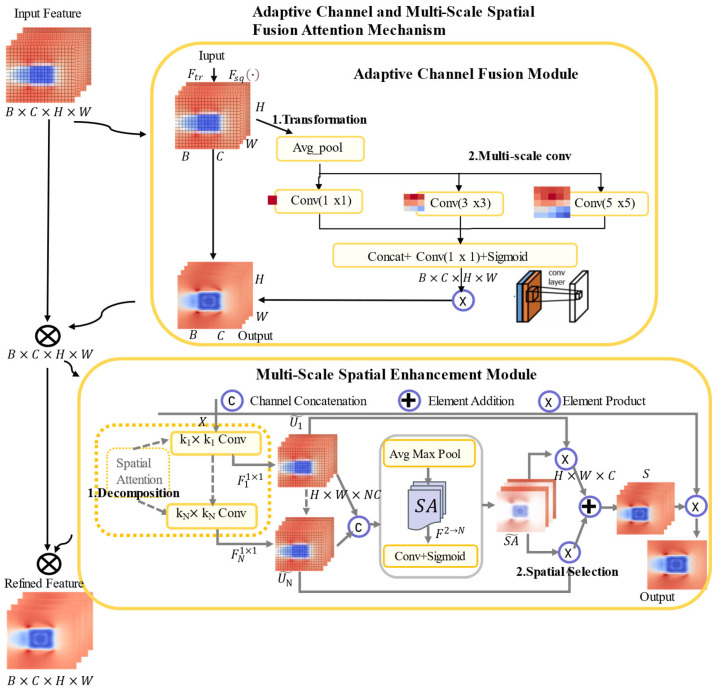
CSFAM architecture.

Through the macroscopic division of labor and synergy between these two modules, CSFAM can precisely address the parameter sensitivity and spatial complexity arising from the strong coupling of fire source–incoming wind–building geometry in high-rise building fire wind fields, ultimately producing a wind field distribution that more closely follows physical principles. Figure 1 shows the structure of CSFAM.

Given the input feature map **X**, a channel attention mechanism is first applied to refine it, denoted as ChannelWeight(⋅), yielding the intermediate feature **S**. Then, S is passed to the spatial attention branch, denoted as SpatialWeight(⋅), to generate the spatial attention map **Y**. The final output feature $Y_{out}$ is computed based on the original feature **X** and the generated spatial attention map **Y**.

B denotes the batch size. C denotes the number of input feature channels, fixed at 5 in this work. The five channels are: incoming wind speed, incoming wind direction, heat release rate, fire source location, and the area influenced by the wind field, and H and W represent the height and width of the spatial grid, respectively.

The wind-field magnitudes are region-wise averaged to obtain a matrix form, so that at each prediction step the entire matrix can be predicted simultaneously. Through this approach, a single forward pass yields consistent predictions for all positions on the entire H × W spatial grid.

(1) $$S\in {\mathbb{R}}^{B\times C\times H\times W}=X⊙\mathrm{ChannelWeight}\left(X\right)$$

(2) $$Y\in {\mathbb{R}}^{B\times C\times H\times W}=S⊙\mathrm{SpatialWeight}\left(S\right)$$

(3) $$Y_{out}\in {\mathbb{R}}^{B\times C\times H\times W}=Y+X$$

#### 2.2.2. Adaptive Channel Fusion Module

The Adaptive Channel Fusion Module adaptively learns the significance of each channel by assigning weights, thereby enhancing salient features while suppressing less relevant information. In this study, the original fully connected layers are replaced by multi-scale convolutional layers employing kernels of varying sizes to extract multi-scale channel correlations, thus enabling localized channel modeling.

This module is designed to address the highly unbalanced channel importance characteristic by adaptively learning the weight of each channel, thereby reinforcing salient features strongly associated with the fire wind field while suppressing noise or secondary channels. This enhances the model’s feature representation capability in complex multi-physics coupled environments and avoids the prediction bias caused by traditional fully connected layers treating all global channels equally.

In the multi-scale convolution stage, 1 × 1, 3 × 3, and 5 × 5 kernels are employed to first expand and subsequently reduce the channel dimensions. A nonlinear sigmoid function generates a weight vector, effectuating parameterized gating. This weight vector is multiplied element-wise with the input feature map to emphasize effective channel features while suppressing irrelevant information. The multi-scale convolution kernels operate independently to capture rich feature interactions and mitigate uneven weight distribution.

The transformation aggregates features **U** along the spatial dimensions of shape B × C × H × W to produce the channel descriptor **Z**, which captures global spatial information. $F_{\mathrm{sq}}()$ denotes the squeeze operation via global average pooling. The squeeze operation condenses the global channel information from the near-fire-source region and the far-field region, avoiding deviations in subsequent convolutions due to local noise.

(4) $$Z=F_{\mathrm{sq}}\left(U\right)$$

In high-rise fire wind fields, small-scale and large-scale features coexist near the fire source. Utilizing convolutional kernels of varying scales for spatial selection enables the model to capture correlations within spatial regions. Initially, the input feature $Z\in {\mathbb{R}}^{B\times C\times H\times W}$ is processed through an average pooling layer $\mathrm{AvgPool}\left(\cdot \right)$ to obtain Y. Then, different scale convolutional layers are applied to Y to derive $\mathrm{conv}1\left(Y\right)$, $\mathrm{conv}2\left(Y\right)$, and $\mathrm{conv}3\left(Y\right)$ with varying receptive fields, which are concatenated $\mathrm{Concat}\left(\cdot \right)$ as follows:

(5) $$Y\in {\mathbb{R}}^{B\times C\times H\times W}=\mathrm{AvgPool}\left(Z\right)$$

(6) $$Y\in {\mathbb{R}}^{B\times C\times 1\times 1}=\mathrm{Concat}\left(\mathrm{conv}1\left(Y\right),\mathrm{conv}2\left(Y\right),\mathrm{conv}3\left(Y\right)\right)$$

The multi-scale convolution $\mathrm{conv}\left(\cdot \right)$ operation first processes the descriptor $Y\in {\mathbb{R}}^{B\times C\times H\times W}$ through multiple convolutional layers and subsequently obtains the corresponding weight $\mathrm{sigmoid}\left(\cdot \right)$ representation $S\in {\mathbb{R}}^{B\times C\times H\times W}$ via a gating mechanism. This step targets the dynamic changes in channel importance in the fire wind field, generating parameterized gating to achieve precise emphasis.

(7) $$S=\mathrm{sigmoid}\left(\mathrm{conv}\left(Y\right)\right)$$

Finally, the weights are multiplied with the input features U, thereby emphasizing or suppressing each channel. For the concatenated S, a sigmoid gating function is applied to obtain the weight representation $Y_{out}$ corresponding to each scale feature, and the acquired weights $Y_{out}$ are multiplied with the initial X:

(8) $$Y_{out}\in {\mathbb{R}}^{B\times C\times H\times W}=X⊙S⊙U$$

#### 2.2.3. Multi-Scale Spatial Enhancement Module

Accurate wind field prediction requires models that dynamically adjust multi-scale receptive fields, balancing local details and broad dependencies. Multi-scale large convolution kernels initially extract features at varying receptive fields, followed by adaptive spatially weighted fusion according to the spatial distribution of input features.

- Large Convolution Kernel Decomposition:

To enable adaptive modeling of diverse high-rise fire wind fields, large convolution kernels are factorized into depth-wise convolutions with varying dilation rates. In high-rise fire scenarios, small-scale features near the fire source coexist with large-scale features in the far field; therefore, the decomposition design can dynamically match different spatial scales, avoiding parameter explosion and overfitting caused by a single large kernel. Specifically, let the size of the i-th Depth-wise convolution kernel be k, the dilation rate be d, and the receptive field be RF, defined as:

(9) $$RF_{1}=k_{1},RF_{i}=d_{i}\left(k_{i}-1\right)+RF_{i-1}$$

The module adopts a fixed N = 2 dual-branch parallel design to capture spatial contexts at different scales. Branch 1 is a 5 × 5 depthwise convolution, Branch 2 is a 7 × 7 depthwise convolution. The outputs of the two branches are compressed to dim/2 via 1 × 1 convolutions, concatenated, and then global average pooling and global max pooling produce a 2-channel feature. Adaptive weights are generated by a 7 × 7 convolution to realize branch-weighted fusion, finally mapped back to the dim channels via a 1 × 1 convolution.

Decomposing large convolution kernels into multi-layer convolutions efficiently produces multi-receptive-field features, while greatly reducing parameters. The resulting multi-layer convolutions act on input $X\in {\mathbb{R}}^{C\times H\times W}$ to capture multi-scale contextual information. where $F_{i}^{\mathrm{dw}}()$ is the i-th depth-wise convolution layer with kernel size $k_{i}$ and dilation rate $d_{i}$, and $U_{i}\in {\mathbb{R}}^{C\times H\times W}$ is the output feature representation of the i-th depth-wise convolution layer.

(10) $$U_{0}=X,U_{i}=F_{i}^{\mathrm{dw}}\left(U_{i-1}\right)$$

Assuming there are N decomposed kernels, the output of each kernel is further modeled for inter-channel correlations using a 1×1 convolution layer $F^{1\times 1}\left(\cdot \right)$. Here, ${\tilde{U}}_{i}\in {\mathbb{R}}^{C\times H\times W}$.

(11) $${\tilde{U}}_{i}=F_{i}^{1\times 1}\left(U_{i}\right),i\in \left\{1,\;2\right\}$$

- Spatial Kernel Selection:

To enable the model to focus on the most relevant spatial regions, a spatial selection mechanism is used to perform spatial selection on feature maps of different scales. First, multiple feature maps of varying scales are concatenated along the channel dimension, and then global average pooling $\mathrm{AvgPool}\left(\cdot \right)$ and global max pooling $\mathrm{MaxPool}\left(\cdot \right)$ are applied along the channel direction of $\tilde{U}$ to compress the global channel information:

(12) $$\tilde{U}\in {\mathbb{R}}^{2C\times H\times W}=\mathrm{Concat}\left({\tilde{U}}_{1},{\tilde{U}}_{2}\right)$$

To allow for information interaction among different spatial descriptors, the spatial aggregated features are concatenated, and a convolution layer $F^{2\to N}\left(\cdot \right)$ is used to transform the pooled features with two channels into a spatial attention map with N channels:

(13) $$\hat{SA}\in {\mathbb{R}}^{N\times H\times W}=F^{2\to N}\left(\mathrm{Concat}\left(\mathrm{AvgPool}\left(\tilde{U}\right),\mathrm{MaxPool}\left(\tilde{U}\right)\right)\right)$$

For each spatial attention map $\hat{SA_{i}}\in {\mathbb{R}}^{1\times H\times W}$, a sigmoid gating function is applied to obtain the weight representation corresponding to each scale feature $\hat{SA_{i}}\in {\mathbb{R}}^{1\times H\times W}$. The N weights are then used to perform a weighted sum with the features from N scales, and finally, the attention features $S\in {\mathbb{R}}^{C\times H\times W}$ are obtained through the convolution layer $F\left(\cdot \right)$.

(14) $$S_{i}\in {\mathbb{R}}^{C\times H\times W}=\mathrm{sigmoid}\left(\hat{{SA}_{i}}\right)⊙{\tilde{U}}_{i}$$

(15) $$S\in {\mathbb{R}}^{C\times H\times W}=F\left(S_{i}+\cdots +S_{N}\right)$$

The final output of the spatial weighting module is the element-wise product between the input features $X\in {\mathbb{R}}^{C\times H\times W}$ and S. The output $Y\in {\mathbb{R}}^{C\times H\times W}$ is the final output of the spatial weighting module.

(16) $$Y\in {\mathbb{R}}^{C\times H\times W}=X⊙S$$

### 2.3. MLP Application in Wind Field Prediction

In high-rise fire wind field prediction, the MLP effectively captures complex variations in wind speed and direction along the vertical profile through its multilayer nonlinear mapping capabilities. Thermal disturbances generated by the fire source induce vertical updrafts and lateral perturbations within the atmospheric boundary layer. The MLP models vortex structures and thermal convection through multiple activation layers, approximating local airflow characteristics in the fire environment. To address spatial heterogeneity of the wind field, the MLP incorporates building structures and terrain information, modeling wind field enhancement and guidance effects near the fire, which critically influence fire spread rate and fire line direction.

Thermal winds and flame-induced updrafts substantially influence the upper-level wind field. The MLP’s multilayer architecture learns these nonlinear relationships, enabling accurate prediction of high-altitude wind field variations in fire scenarios. By incorporating historical data, the MLP captures wind field evolution patterns, particularly adapting to rapid changes during fire outbreaks. It predicts both vertical and horizontal wind fields while reflecting characteristic wind types, such as local eddies and flame-induced winds.

This work adopts a supervised regression task with the goal of predicting the distribution of the horizontal wind-speed magnitude on a two-dimensional plane in a fire scenario from multiple physical input conditions. The input is a tensor of shape (B, 5, 37, 37), consisting of five normalized physical channels: inflow wind speed, inflow wind direction, heat release rate, fire source location, and the area influenced by the wind field; the output is a single-channel predicted field of shape (B, 1, 37, 37), representing the normalized wind-speed magnitude.

The MLP module first enhances the input features via CSFAM (channel and spatial attention), then flattens to a 6845-dimensional vector (5 × 37 × 37 = 6845), which is mapped through four fully connected layers (6845 → 512 → 256 → 128 → 1369) and reshaped back to (B, 1, 37, 37).

### 2.4. Time Complexity and Mechanism Analysis of CSFAM

To quantitatively assess the computational efficiency of the proposed CSFAM, this section begins with an analysis from the perspective of time complexity. The input feature map is denoted as $X\in {\mathbb{R}}^{B\times C\times H\times W}$, where B denotes the batch size, C = 5 is the fixed input channel number corresponding to the incoming flow wind speed, wind direction, heat release rate, and fire source position, and H and W are the spatial grid resolutions. CSFAM’s channel module compresses the spatial dimensions via global average pooling, then performs parallel 1 × 1, 3 × 3, and 5 × 5 multi-scale convolutions on the reduced descriptor. As the spatial size is already compressed, this step introduces only negligible $O\left(B\cdot C^{2}\right)$, while the Sigmoid gating and element-wise rescaling contribute $O\left(B\cdot C\cdot H\cdot W\right)$ again. The spatial module adopts a dual-branch depth-separable convolution with 5 × 5 and 7 × 7 kernels, substantially reducing the standard convolution overhead to $O\left(B\cdot C\cdot H\cdot W\cdot 80\right)$, with the overall CSFAM operation amounting to O(B⋅C⋅H⋅W⋅κ), where κ is a small constant independent of the input resolution. Since C⋅H⋅W is small, this complexity simplifies to a linear form in the batch size O(B), on the same order of magnitude as the baseline MLP, with CSFAM introducing only a negligible constant factor overhead.

Viewed from the intrinsic mechanism of CSFAM, its lightweight design fundamentally circumvents the high computational barriers of traditional CFD and CNN, realizing CPU time compression. The channel module thoroughly decouples the dense channel interactions from the full spatial convolution in CNN via global pooling plus spatial linear transformation, avoiding parameter inflation and memory access costs on high-dimensional feature maps; the spatial module employs depth-separable convolution and multi-scale adaptive weighting, targeting only the fusion of local patterns associated with physical priors rather than global traversal. Under the synergistic effect of the aforementioned mechanisms, while enhancing prediction accuracy, CSFAM directly compresses the CPU time from the hour-scale of CFD to the second-scale, breaking the computational bottleneck in real-time fire warning.

## 3. Dataset and Set-Up

This section summarizes the procedures of numerical simulations, and mesh generation, along with multiple metrics for assessing predictive performance.

### 3.1. CFD Simulation

This section details the procedures for numerical simulation, and mesh generation. For numerical simulation, CFD simulations employing the RANS approach were conducted.

#### 3.1.1. RANS Trade-Offs and Validation

The LES approach is capable of directly resolving large-scale turbulent structures, thereby capturing more accurately the transient complex phenomena in fire-induced wind fields, including local recirculation zones, the dynamic interactions between fire plumes and incoming winds, and vortex-dominated structures. In contrast, RANS treats turbulence through time averaging, solving only the averaged momentum and energy equations and employing the eddy-viscosity hypothesis to close the Reynolds stresses.

This study adopts the RANS scheme for the simulation of fire-induced wind fields. Under the constraints of existing computational resources and time, generating 1050 large-scale datasets covering a variety of incoming wind speeds, wind directions, and fire source conditions necessitates the selection of the RANS scheme, which has a lower computational cost than LES.

Although RANS possesses advantages in computational efficiency, it still exhibits certain limitations: (1) its resolution capability for strongly separated flows, recirculation zones, and complex vortex structures in wakes is relatively weak, potentially leading to an overestimation of local turbulent kinetic energy; (2) its capture of transient pulsations in fire plume–wind interactions is relatively limited, which may result in the smoothing of some high-frequency disturbances. The primary impact of these limitations on this study lies in the fact that the training datasets generated by RANS may exhibit certain deviations in local recirculation and vortex structure regions, thereby causing a certain degree of smoothing effect in the CSFAM-MLP model when capturing high-frequency details.

To further quantitatively assess the correlation between the RANS and LES methods this study selected representative sample cases that cover as wide a parameter space as possible. The following Table 2 summarizes the Pearson correlation coefficient values for each region under three typical wind speed levels small wind speed 1 m per second medium wind speed 3 m per second large wind speed 5 m per second as well as two inflow directions 0 degrees and 45 degrees.

(17) $$r=\frac{\sum_{i=1}^{n}\left(x_{i}-\bar{x}\right)\left(y_{i}-\bar{y}\right)}{\sqrt{\sum_{i=1}^{n}{\left(x_{i}-\bar{x}\right)}^{2}\sum_{i=1}^{n}{\left(y_{i}-\bar{y}\right)}^{2}}}$$

The Pearson correlation coefficient is r, where $x_{i}$ and $y_{i}$ are respectively the i-th observation values of the two variables, $\bar{x}$ and $\bar{y}$ are respectively the sample means of the two variables, and *n* is the sample quantity.

According to the results in the Table 2, although the RANS model’s simplification of local recirculation, plume interactions, and vortex-dominated structures prevents it from resolving unsteady vortex structures in the same manner as LES, the results indicate that the RANS predictions of the average wind field in the windward and side regions are relatively consistent with those of LES.

In summary, these Pearson correlation coefficients remain within the engineering-acceptable range and are superior to the thresholds established in existing standards and papers. In CFD validation studies of building wind environments and pollutant dispersion, a Pearson correlation coefficient greater than 0.8 is typically regarded as the acceptance criterion for good consistency. In the present study, the Pearson correlation coefficient is lowest at 0.832 and highest at 0.922; both are superior to this threshold.

#### 3.1.2. Theoretical Basis for CFD

This study referred to the authoritative best practice guidelines proposed by the Architectural Institute of Japan and confirmed the applicability of the settings for the statistical average wind speed field of primary interest.

The solver type was set to a steady-state solver, the discretization scheme adopted the finite volume method, and the pressure-velocity coupling was realized through the SIMPLEC algorithm. The convergence criterion was that the residuals of all calculations were below $10^{-4}$.

This study employed the steady-state Reynolds-Averaged Navier–Stokes (RANS) method and used the standard k − ε turbulence model as the primary closure model. This modeling assumption was physically reasonable: in the high-rise building fire wind field, the incoming wind speed was stable, the temperature rise in the fire region led to a reduction in local air density, thereby forming a statistically steady mean wind speed field. These mean wind speed distributions were exactly the core object of prediction in this study. The steady-state RANS solved the mean momentum and energy transport processes through time-averaged equations and adopted the eddy-viscosity assumption to close the turbulent Reynolds stresses. It could reliably capture the aforementioned mean wind speed field characteristics at a reasonable computational cost and was particularly suitable for generating large-scale datasets to train the subsequent CSFAM for engineering wind speed magnitude prediction.

In this study, the inlet boundary condition adopted Velocity Inlet combined with Vector Inlet to specify the incoming wind speed magnitude and direction. This setting physically corresponded to the stable incoming wind field in the atmospheric boundary layer and could accurately drive the wind speed distribution around the high-rise building. The outlet adopted the Pressure Outlet condition, which allowed the downstream flow field to develop naturally without artificial reflections. The building and ground surfaces were set as impermeable walls, with conjugate heat transfer (CHT) enabled at the fluid-solid interfaces to account for thermal interactions between the hot fire-induced flow and the building structure.

This study preferentially adopted a hex-dominant hybrid meshing strategy, in which polyhedral elements were mainly used in the core flow-field region to balance the adaptability to complex geometries and the overall computational economy. To accurately simulate the near-wall heat loads and wind load distributions, special emphasis was placed on controlling the y+ values in the near-wall region, thereby achieving consistent and high-quality boundary layer resolution.

#### 3.1.3. Numerical Simulation and Computational Domain

The parameter settings of this study referenced the experimental settings from the paper Effect of wind speed and direction on facade fire spread in an isolated rectangular building and the parameter settings from the paper Influence of Wind Direction on Fire Spread on High-Rise Building Facades, with the adopted experimental parameters fully encompassing the parameter ranges of the aforementioned literature.

The primary influencing factors in the dataset include incoming wind speed, incoming wind direction, fire source location, and fire source size. To determine the initial wind speed for the urban wind field, different levels of wind speeds were selected according to the wind force grading standards established by the China Meteorological Administration. Due to the symmetry of the building, five wind directions ranging from 0 to 90 degrees were chosen as the initial wind directions. The fire intensity ranges from small to large, encompassing a wide array of potential fire intensities. Table A1 in Appendix A is excerpted from the Technical Standard for Building Smoke Control and Exhaust System. The experimental parameter settings are based on this standard.

**Figure 2:**
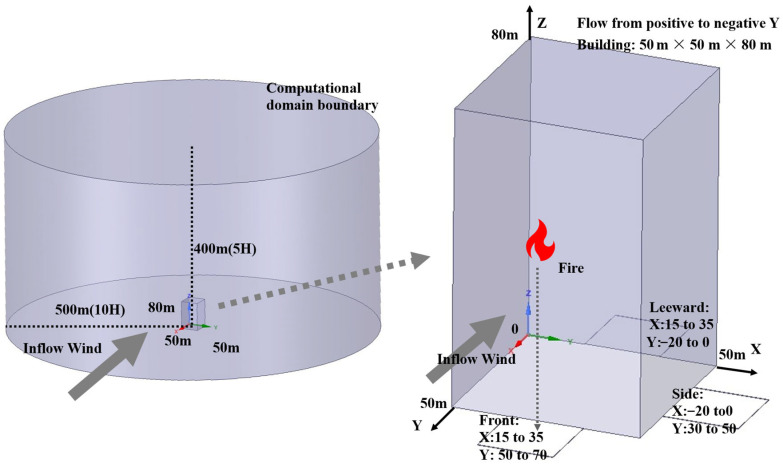
Building modeling coordinate diagram.

In the CFD numerical simulation section, the overall model design is shown in Figure 2. A high-rise building model with dimensions 50 m × 50 m × 80 m (L × W × H) is placed in a wind field with a vertical wind-speed gradient. The computational domain is cylindrical, with a height of 400 m and a radius of 500 m. The radius of the computational domain is approximately 10 H the building’s length and width, and the height of the computational domain is approximately five times the building height. The specific parameters used in this study’s numerical simulations are listed in Table 3.

Figure 2 Schematic diagram of the building modeling coordinate system adopted in the CFD simulation of fire-induced wind fields around an isolated rectangular high-rise building. The coordinate origin is located at the building apex (0, 0, 0). The x- and y-axes align respectively with the building length and width of 50 m, while the *z*-axis points vertically upward to the building height of 80 m. All coordinate axes, the origin, building dimensions, and key reference markers of the computational domain are explicitly annotated in the figure.

The heat source settings in this study refer to the SFPE Handbook of Fire Protection Engineering, the Technical Standard for Smoke Prevention and Exhaust Systems in Buildings, and the Shanghai Standard for Design of Smoke Prevention and Exhaust Systems in Buildings. Within commonly used heat source ranges, different heat release rates (HRRs) were adopted.

The building settings of this study referenced the Unified Standard for Civil Building Design and the Code for Urban Residential Area Planning and Design. The wind speed and wind direction settings referenced the wind scale standard issued by the China National Standardization Management Committee. The heat source settings referenced the SFPE Handbook of Fire Protection Engineering, the Technical Standard for Smoke Management Systems in Buildings, and the Shanghai Design Standard for Smoke Management Systems in Buildings, while different heat release rates were adopted within the range of commonly used heat sources.

In this study, a total of 1050 high-rise wind-field samples were generated through computational fluid dynamics (CFD) simulations. The dataset was partitioned according to the fire source heat release rate (HRR) as follows: a training set consisting of samples with fire source powers of 0 MW, 5 MW, and 10 MW; a validation set consisting of samples with a fire source power of 2.5 MW; and a test set consisting of samples with a fire source power of 7.5 MW. This partitioning strategy is intended to enable a rigorous assessment of the model’s extrapolation capability and robustness to out-of-distribution inputs.

The 1050 CFD cases correspond to 1050 unique combinations of inflow wind conditions and fire-source states. For each combination, wind-field distributions were systematically extracted at 100 vertical heights spaced 1 m apart, resulting in an effective dataset of 105,000 wind-field planes.

The inflow wind direction at 0° is along the positive *Y*-axis, pointing toward the negative *Y*-axis, as shown in the figure. Define the windward side range as *X*-axis from 50 m to 70 m and *Y*-axis from 15 m to 35 m. The side range is defined as *X*-axis from 30 m to 50 m and *Y*-axis from −20 m to 0 m. The leeward side range is set as *X*-axis from −20 m to 0 m and *Y*-axis from 15 m to 35 m. The sampling plane is fixed in the global coordinate system, with the windward, crosswind, and leeward faces all defined as 0° relative to the initial wind direction, and they remain unchanged during changes in wind direction. The origin is at one corner of the building, with the opposite diagonal coordinates being (50, 50).

#### 3.1.4. Fluid Properties

In this study, high-rise fire wind field simulations were conducted using standard thermodynamic properties of air, including a density of 1.2 kg/m^3^, specific heat capacity of 1005 J/(kg·K), thermal conductivity of 0.0257 W/(m·K), and kinematic viscosity of approximately 1.5 × 10^−5^ m^2^/s. The fire source was positioned on the building surface.

#### 3.1.5. Grid Independence Analysis

After mesh generation, the built-in mesh quality diagnostic module in the CFD software was used to perform a comprehensive check and processing of key indicators such as cell skewness. Furthermore, to verify the mesh independence with respect to grid resolution, this study further adopted the Grid Convergence Index (GCI) method, in which $v_{i}$ represents the area-averaged wind speed in the grids near the building, the grid refinement ratio is r, assuming a second-order accuracy p of 2.0 and a safety factor F of 1.25, ultimately obtaining a fine-grid GCI of approximately 2.6%.

(18) $$\mathrm{GCI}=\frac{F\times \left|\frac{v_{2}-v_{1}}{v_{1}}\right|}{r^{P}-1}\times 100\%$$

Table 4 shows that, as the grid is continuously refined, the GCI values exhibit a clear monotonic decreasing trend, indicating that the grid scheme has achieved a good convergence state. The final fine-grid GCI is approximately 2.6%. This value indicates that, when generating large-scale machine learning training datasets rather than pursuing a single high-precision prediction, the discretization uncertainty of the selected 0.4 m grid has been controlled within an acceptable level of 5% to 10%, meeting the engineering application requirements.

### 3.2. Real-World Scene Data

To verify the consistency between the wind field generated by CFD and real-world conditions, this study conducted the following systematic verification process. First, a similar high-rise building model was selected, with a length of 50 m, a width of 50 m, and a height of 80 m; the actual size deviation was controlled within ±3 m to ensure consistency with the geometric dimensions used in the CFD simulation.

The measurement campaign was conducted under multiple clear and stable atmospheric conditions over several days. From the full dataset, approximately 200,000 valid point samples were randomly selected, covering wind speeds in the range of 0–7 m/s and a variety of typical wind directions.

Wind speed measurements were performed using an anemometer, with a typical uncertainty of ±0.3 m/s. The measurement points were located at the centers of the four sides of the target building, at heights of 0.5 m, 10 m, 30 m, 50 m, and 70 m, respectively, corresponding to the CFD sampling positions. At the same time, a reference anemometer was installed in the adjacent open and flat terrain to record the inflow conditions. A two-point synchronous measurement strategy was adopted: the wind speed at the reference point was directly used as the inflow boundary condition for the corresponding CFD case, while the measured wind speed and wind direction at the target building points were directly compared with the CFD predicted values. Table 5 provides a detailed description of the real-world measurement parameters.

The typical measurement uncertainty of the anemometer is ±0.3 m/s. In the comparison process, an error range of ±0.3 m/s was introduced for each measurement point, and a sensitivity analysis was performed using the inflow data recorded by the reference anemometer. The Proportion of Points within ±0.3 m/s Interval is calculated as the percentage of verification points at each height for which the absolute deviation between the CFD-predicted wind speed and the measured wind speed does not exceed ±0.3 m/s, relative to the total number of verification points at that height.

The point-to-point comparison results show that the deviations between the CFD predicted values and the measured values at verification points of different heights mostly fall within the ±0.3 m/s measurement uncertainty interval, as detailed in Table 6. Specifically, at the intermediate heights of 30 m and 50 m, the Mean Relative Error is the lowest, at 6.2% and 5.5% respectively, and the proportions of points falling within the interval are 88.2% and 90.1% respectively; at the near-ground height of 0.5 m, the Mean Relative Error is 14.5%, and the proportion of points falling within the interval is 78.4%; at the high-altitude height of 70 m, the Mean Relative Error is 7.8%, and the proportion of points falling within the interval is 85.6%. These quantitative indicators indicate that the CFD predicted values have good consistency with the characteristics of the measured wind field.

## 4. Results and Discussion

This section presents an analysis of wind field data alongside neural network prediction results. Wind field variations around high-rise buildings are pronounced, with the fire source exerting a significant influence on the windward façade. The CSFAM model achieves a mean squared error (MSE) of 0.0004, demonstrating superior performance relative to other models.

### 4.1. Dataset Result Analysis

The following analysis indicates that the wind field exhibits different functional characteristics across various regions, showcasing distinct variations.

#### 4.1.1. The Relationship Between Wind Speed and Height

**Figure 3:**
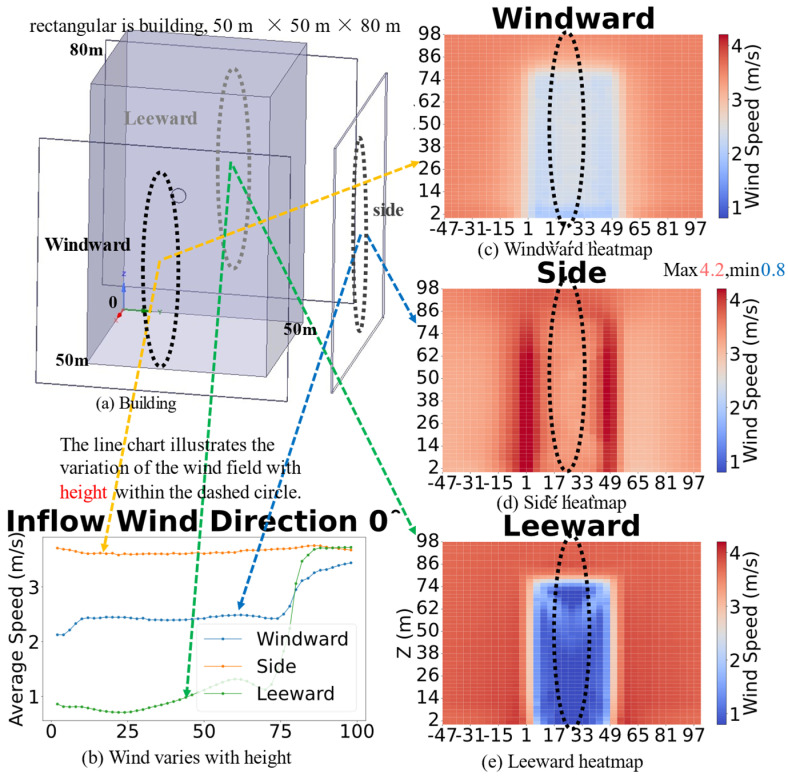
The impact of height on regional wind field.

Figure 3 Wind speed heat maps around the isolated rectangular high-rise building base dimensions 50 m × 50 m, height 80 m and the variation in wind speed with height as a functional relationship, under an inflow wind direction of 0°. The coordinate origin is located at the building apex (0, 0, 0). The heat maps of wind speed on the windward, side, and leeward faces are shown; the line plot displays the variation in average wind speed with height. All coordinate axes, building extents, monitoring lines, and key features are explicitly annotated in the figure.

On the windward façade, wind speed is very low at 0–5 m, increases sharply to approximately 0.5 m/s between 5 and 10 m, remains stable from 10 to 80 m, and gradually approaches the inflow wind speed above 80 m, indicating a greater influence of external wind at higher altitudes. On the lateral façade, wind speed remains relatively constant with no significant fluctuations; the heatmap reveals that the corners are sensitive regions, although they are less affected by the inflow wind speed. On the leeward façade, wind speed increases gradually with height, decreases slightly at 60–70 m, and then rises rapidly to converge with the inflow wind speed above 70 m, indicating a significant increase in wind speed at higher elevations.

#### 4.1.2. Comparison of Heat Maps with and Without Heat Sources

**Figure 4:**
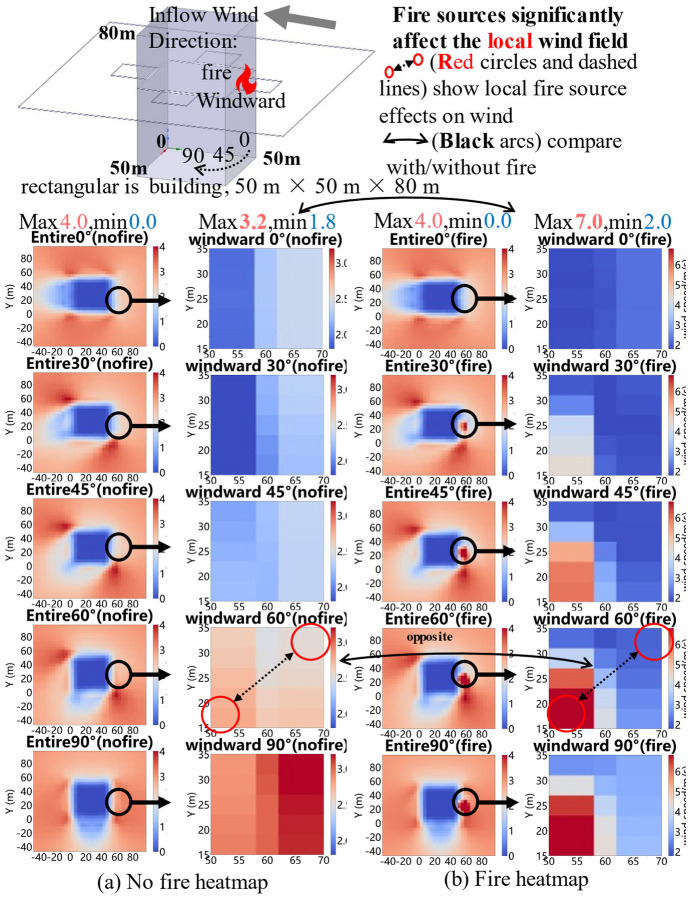
Comparison heatmap with and without fire source.

The wind field heat map distributions around the isolated rectangular high-rise building under inflow wind directions of 0–90° with and without fire source are illustrated in Figure 4, with the coordinate system and building dimensions (50 m × 50 m base, 80 m height) consistent with Figure 2.

Figure 4 illustrates the wind field heat map distributions around the isolated rectangular high-rise building under inflow wind directions of 0–90° with and without a fire source. In the absence of the fire source, wind speeds near the building are significantly lower than those in regions farther away. Conversely, the presence of the fire source results in a marked increase in wind speeds near the building, while regions farther from the fire source exhibit lower wind speeds. This contrast underscores the significant role of the fire source in modulating the spatial distribution and intensity of the surrounding wind field in fire simulations, particularly at 60° and 90° wind directions, where the fire-induced local wind speed changes are more pronounced, as indicated by the red circles and the “opposite” labeled areas in the figure.

#### 4.1.3. Statistical Comparison with and Without Heat Sources

**Figure 5:**
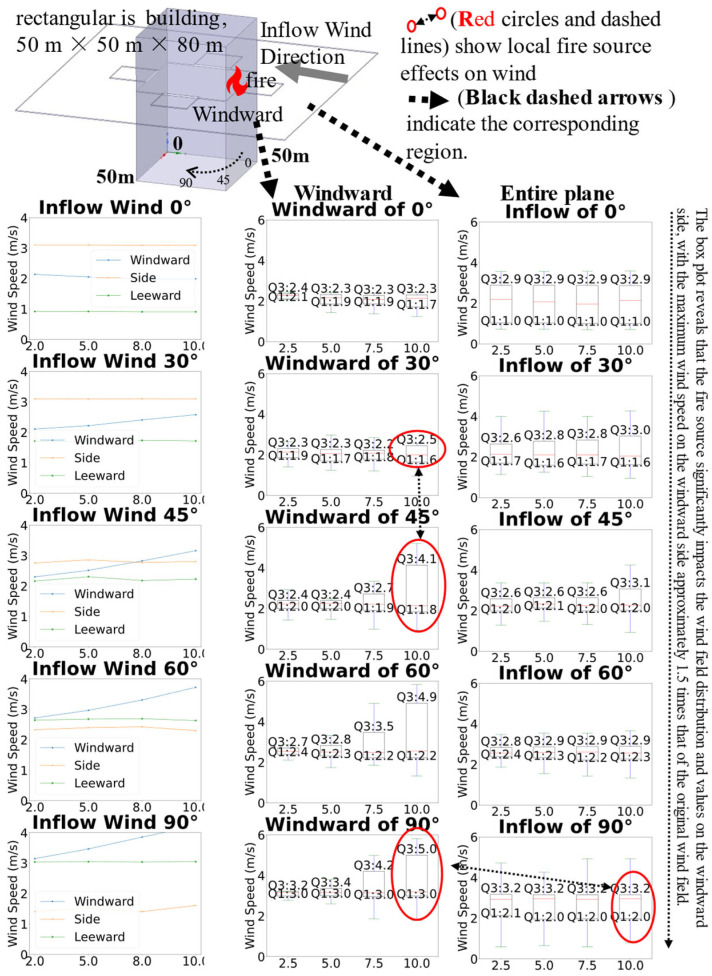
Impact of Wind Direction on the Regional Wind Field.

Figure 5 presents the statistical comparison of average wind speeds with and without heat sources on the windward, side, and leeward faces of the building under 0° and 45° inflow wind directions. At 0° wind direction, the average wind speed on the windward face is approximately 2.10 m/s, higher on the side face, and lowest on the leeward face. This shows typical blunt-body flow features: a stagnation zone forms on the windward face leading to reduced velocity, significant acceleration occurs on the side face due to shear layer effects, and the leeward face lies in a low-speed wake recirculation region.

At 45° wind direction, the windward face wind speed increases to approximately 2.73 m/s, the side face slightly decreases to about 2.82 m/s, and the leeward face clearly rises to about 2.24 m/s. This indicates a transition from direct normal impingement to more oblique flow around the building, with a noticeable shift in the flow separation point location.

Furthermore, to rigorously assess the uncertainty in the results, the following two metrics are adopted:

The core indicator is the Standard Error of the Mean (SEM = Std/√N), which quantifies the sampling uncertainty of the mean wind speed in each region. The auxiliary indicator is the Relative Standard Error (RSE = SEM/Mean) together with an approximate 95% confidence interval (CI).

On the windward face: Std ≈ 0.33–0.37, SEM ≈ 0.074–0.083, RSE ≈ 3.4–4.2%. On the leeward face: Std ≈ 0.134–0.139, SEM ≈ 0.029–0.030, RSE ≈ 3.1–3.3%. These metrics collectively indicate that the uncertainty on the leeward face is relatively lower. The uncertainty levels on the windward and side faces are similar to each other and slightly higher than on the leeward face, but remain within an acceptable range overall. This allows reliable cross-region comparison and trend inference.

#### 4.1.4. The Impact of Fire Source Height on Wind Field

**Figure 6:**
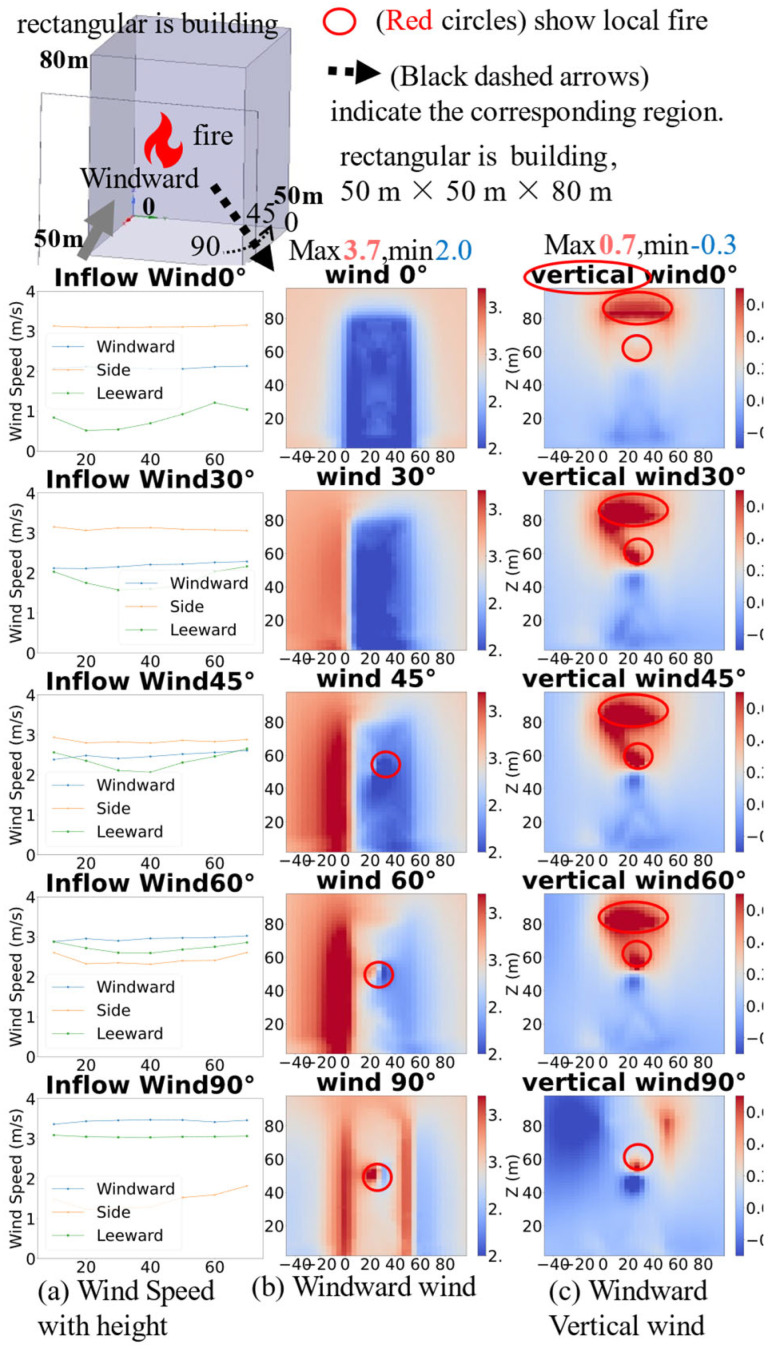
The impact of height on regional wind field.

Figure 6 shows that, under different wind direction angles, the wind-speed distributions on the three façades of a high-rise building exhibit significant directional dependence and asymmetry. At a wind direction of 0°, the average wind speed on the windward face is about 2.1 m/s, with the leeward face being the lowest, which is consistent with the typical three-dimensional bluff-body around-flow behavior: the windward face forms a stagnation region, the side shear layer accelerates, and the leeward face lies in a low-speed recirculation region. When the wind direction increases to 45°, the wind speed on the windward face rises to about 2.5 m/s, the side faces decrease slightly, reflecting a transition of the flow from direct frontal impact to oblique around-flow, with the separation point moving downstream and the wake asymmetry enhanced. At 60° and 90°, the region of maximum wind speed shifts to the windward face, and the side faces evolve into a new low-speed region.

In the figure, the first column shows the line plots of wind speed variation with height on each plane, the second column presents the wind speed heatmap on the windward face, and the third column displays the vertical wind speed heatmap on the windward face. All panels focus on the windward face, with red circles marking the fire source region, intuitively illustrating the significant impact of changes in fire source height on the wind field distribution of the windward face.

Heat maps show that the high-speed region in front of the windward face concentrates near the stagnation point; the corresponding locations on the building appear as blue low-speed zones, reflecting solid blockage and near-wall velocity deficit. The vertical wind-speed heat map on the right reveals a significant upward plume effect induced by the buoyancy of the fire source, which is particularly strong on the leeward side at low wind speeds, facilitating rapid ascent of hot smoke along the façade. The low-height wind speeds are more strongly influenced by near-ground recirculation and the ground boundary layer; the middle-to-upper portions more clearly reflect the superposition of the free-stream flow and building disturbance; as height increases, the wind-speed gradients on the side and leeward faces gradually diminish.

### 4.2. Analysis of Neural Network Results

This section evaluates the performance of the Channel and Spatial Feature Attention Module (CSFAM). Ablation studies demonstrate that CSFAM achieves a mean squared error (MSE) of 0.0004, outperforming both MLP and CBAM. In comparative experiments, CSFAM-MLP achieves an MSE of 0.0004 and a mean absolute error (MAE) of 0.0141, outperforming all other tested models.

#### 4.2.1. Model Evaluation

During data preprocessing, the mean squared error (MSE) was chosen as the loss function to quantify the difference between the model’s output and the actual target values. The MLP employs a four-layer fully connected architecture, trained for 80 epochs with a learning rate of 0.0003 and optimized using the Adam algorithm.

#### 4.2.2. Comparison of Prediction Models

This study selected various attention mechanisms—including Squeeze-and-Excitation Networks (SENet), LSKnet, CBAM, Exponential Moving Average (EMA), Add, and ReLU—and combined them with five distinct neural network architectures: AlexNet, LeNet-5, Multilayer Perceptron (MLP), ResNet, and VGG16. Extensive comparative experiments were conducted to validate the effectiveness of these combinations. A total of 32 neural network models were evaluated, with a focus on their performance across key metrics such as Mean Squared Error (MSE), Mean Absolute Error (MAE), Symmetric Mean Absolute Percentage Error (SMAPE), and other related performance indicators. The results indicate that CSFAM achieves very high accuracy in the domain of wind-field prediction for high-rise fires, underscoring its superiority.

In summary, CSFAM not only surpasses all comparative models across key indicators but also integrates Adaptive Channel and Multi-Scale Spatial Fusion Attention Mechanism, effectively enhancing feature representation and information fusion. This comprehensive performance comparison clearly illustrates CSFAM’s ability to significantly improve prediction accuracy, particularly in scenarios characterized by high data complexity and rapid variability. Its outstanding performance offers new avenues for future research and practical applications, establishing a solid foundation for technological advancement in related domains. Table 7 presents a detailed overview of the comparative experiment results.

#### 4.2.3. Ablation Experiment

In this study, ablation experiments were conducted to assess the effectiveness of the CSFAM neural network in wind field data prediction. Four configurations were evaluated: (1) no attention mechanism; (2) Adaptive Channel Fusion Module only; (3) Multi-Scale Spatial Enhancement Module only; and (4) the complete CSFAM structure. Table 8 summarizes the experimental results for each configuration. As shown in the table, CSFAM demonstrates clear advantages across all performance metrics, indicating its effectiveness in enhancing overall model performance. The standard deviation refers to the standard deviation of the population.

#### 4.2.4. Comparison of Convolutional Layers in the Adaptive Channel Fusion Module

The multi-scale fusion version of the adaptive channel fusion module employs parallel integration of 1 × 1, 3 × 3, and 5 × 5 convolutions. Under the modest constraint that the parameter count increases by only approximately 6% compared to the largest single-scale version and by about 22% compared to the smallest single-scale version, this design achieves the highest R^2^, the lowest MAE and MSE on the test set, as well as the best sample-to-sample prediction consistency. In particular, for the velocity field regression task, the multi-scale design significantly enhances the model’s comprehensive capability to capture point-wise dependencies, local vortex structures, and large-scale wind-field guiding features within the wind-fire coupling field. Overall, both prediction accuracy and stability are clearly superior to those obtained with single-scale implementations.

Table 9 presents the performance comparison for varying parameter counts, with the channel module portion set to channel = 5 and the performance metrics taken as the best values observed in the later stage of training.

#### 4.2.5. Comparison of Baseline Neural Networks

This study selects AlexNet, VGG, ResNet, MLP, LeNet, DenseNet, and ViT as baseline neural networks. Analysis indicates that network depth and architecture are crucial for feature extraction from wind-field data. For MLP, with relatively few parameters and a simple structure, converging quickly and capturing information effectively, a good performance is achieved with an MSE of 0.0089. ViT, leveraging a self-attention mechanism, significantly improves performance, with an MSE of 0.0081, MAE of 0.0716, and SMAPE of 3.6482, and it excels at capturing long-range dependencies and complex spatiotemporal patterns. By comparison, ResNet performs moderately, with an MSE of 0.1755. Table 10 presents a performance comparison among the networks.

The current baseline results delineate the upper limit of architectural accuracy achievable for this task, while simultaneously revealing the systematic limitations inherent in the conventional computer vision transfer learning paradigm when applied to this domain. The strong performance of CSFAM-MLP demonstrates that designing specialized attention mechanisms tailored to fluid physical fields (particularly fire-induced wind fields), capturing multi-physical coupling along the channel dimension while focusing on dynamically critical regions in the spatial dimension—constitutes a core and promising direction for unlocking further potential. To achieve more robust and higher-precision predictions under higher-resolution grids, more complex fire source conditions, and realistic multi-building urban scenarios, it is necessary to incorporate adaptive channel attention together with multi-scale spatial fusion attention mechanisms.

## 5. Conclusions

This study innovatively proposes an Adaptive Channel and Multi-Scale Spatial Fusion Attention Mechanism (CSFAM), combined with a Multi-Layer Perceptron (MLP) neural network, which effectively addresses the trade-off between prediction accuracy and modeling cost in wind field prediction around high-rise buildings. The approach is built upon a diverse wind field dataset consisting of a total of 1050 cases generated from CFD simulations.

This research not only provides an efficient and reliable tool for urban planning, meteorological monitoring, and building safety management, but also promotes innovative applications of deep learning in the field of fluid dynamics prediction. The introduction of CSFAM significantly enhances the model’s capability to represent detailed features in wind field environments, thereby improving prediction robustness and adaptability to complex scenarios.

The structured dataset, comprising 1050 unique parameter combinations across 100 height planes for a total of 105,000 effective planes, has already provided sufficient coverage for the scalar wind-speed magnitude prediction task.

Ablation experiment results demonstrate that simultaneously incorporating both the Channel module and the Spatial module can substantially improve model performance: MSE is reduced to 0.0004, MAE decreases to 0.0141, SMAPE drops to 0.7219, R^2^ increases to 0.9766, and the prediction standard deviation is significantly lowered to 0.182. These results clearly reveal a strong synergistic gain effect between the two modules, markedly outperforming models that use either single-channel or single-spatial attention alone.

Furthermore, this study relies entirely on data-driven methods and does not introduce explicit physical constraints, such as mass conservation or momentum conservation. The impact of these violations on the scalar wind speed magnitude prediction task can be regarded as negligible, based on the test set R^2^ of 0.9766, MSE of 0.0004, as well as the real-world validation in which the MSE at each height is below 0.0142.

Although the current model exhibits excellent performance in wind speed prediction, the lack of mass and momentum conservation constraints in purely data-driven models tends to introduce systematic deviations in locally complex flow regions, thereby weakening the model’s ability to capture scalar wind speed trends and consequently reducing physical credibility and long-term prediction stability. Referring to existing literature, data-driven surrogate models without imposed physical constraints can amplify the scalar wind speed magnitude prediction error by 10% compared with PINNs models in complex geometric scenarios.

Future work will explore the physics-informed neural network (PINNs) framework or introduce soft conservation law constraints during the training phase, thereby significantly enhancing the model’s physical consistency and deployment reliability without substantially increasing the computational burden.

Although this study has made notable advancements in predicting fire-induced wind fields in high-rise buildings, several critical limitations remain. As a purely data-driven model, the performance of CSFAM-MLP may be tied to the training dataset. The current dataset focuses on isolated rectangular high-rise buildings with a 50 m × 50 m base and 80 m height, where the model has already shown excellent generalization ability for typical high-rise building types featuring rectangular floor plans and minimal surrounding interference in isolated or semi-isolated configurations. For scenarios with substantially different geometric and environmental conditions, such as irregular high-rises or dense building clusters, the model’s predictions of complex flow structures can be effectively extended through efficient transfer learning. The distinctive lightweight and modular design of CSFAM positions it as an ideal platform for transfer learning: new building types can be rapidly incorporated simply by adding CFD cases, thereby substantially improving the model’s portability and practical utility in real-world urban fire emergency scenarios.

## Figures and Tables

**Table 1:** Recent advances in CFD and ML-CFD methods for wind field prediction.

Research Direction	Research Method	Advantages	Disadvantages	Research Findings
CFD	LES	Able to achieve higher accuracy in results	Additional computational time	Predicting building surface wind fields under varying external wind conditions [24].
	RANS	The most widely used CFD approaches	Unable to simulate transient and unsteady characteristics.	Predicting the circulating airflow within isolated buildings [25].
				High-fidelity prediction of local vortex structures in regions with complex geometries [26]
				Rapid evaluation of wind-field variations across multiple scenarios
ML-CFD	CNN	Easier to train and demonstrate enhanced generalization abilities	Complex structures requiring substantial computational resources	Key characteristics of wind fields under emergency scenarios [27].
				Predicting the distribution of airflow and wind fields across different buildings [28].
				Developing wind-field models incorporating fire sources [29].
				City-scale wind-field assessment [30].
				Developing airflow-field models for neural network training [31].
	Attention Mechanism	Enhancing predictive accuracy and effectively integrating multi-source and multi-scale information.	The approach demands substantial data and entails increased modeling complexity.	Prediction of wind-field distribution using spatial attention [32]

**Table 2:** Correlation between RANS and LES.

Angle	Small Wind Speed	Medium Wind Speed	Large Wind Speed
0°	0.922	0.901	0.878
45°	0.883	0.857	0.832

**Table 3:** CFD simulation settings.

Variable	Data Settings						
Fire source height (m)	10	20	30	40	50	60	70
Heat Release Rate (MW)	0	2.5	5	7.5	10		
Incoming wind speed (m/s)	1	2	3	4	5	6	
Incoming wind angle	0	30	45	60	90		

**Table 4:** Grid Independence Verification Results.

Case	Grid Size (m)	GCI (%)
Case 1	1.0	10.8
Case 2	0.6	7.5
Case 3	0.4	2.6

Note: relative to the baseline finest mesh of 0.2 m.

**Table 5:** Real Data Measurement Parameters.

Parameter Name	Parameter Range	Remarks
Wind Speed	0–7 m/s	Consistent with the wind speed range of the simulation dataset
Direction	0–360°	Within the coverage scope of the simulation dataset.
Altitude	0.5 m, 10 m, 30 m, 50 m, 70 m	Sufficient to meet the requirements for neural network training.
Sampling Rate	1 Hz	The sampling frequency is reasonable.

**Table 6:** Point-to-Point Comparison Key Indicators.

Height (m)	Mean Relative Error (%)	Proportion of Points Within ±0.3 m/s Interval (%)
0.5	14.5	78.4
10	9.8	83.9
30	6.2	88.2
50	5.5	90.1
70	7.8	85.6

**Table 7:** Comparison of prediction models.

NO.	Model Name	MSE ↓	MAE ↓	SMAPE ↓
1	MLP	0.0089	0.0774	3.9733
2	Vit	0.0081	0.0716	3.6482
3	Dense	0.0332	0.1449	7.065
4	Mobilenet	3.549	1.8781	63.6219
5	SEnet-Alex	0.2651	0.2968	13.2842
6	SEnet-lenet	0.0062	0.0729	3.7107
7	SENet-MLP	0.0023	0.0164	0.8638
8	SENet-Resnet	0.0195	0.1042	5.1538
9	SENet-VGG	0.0134	0.0849	4.3343
10	CBAM-Alex	0.2423	0.2563	11.1992
11	CBAM-MLP	0.0031	0.023	1.2102
12	CBAM-Resnet	0.1261	0.2898	13.3682
13	CBAM-VGG	0.013	0.0798	4.0772
14	LSKnet-Alex	0.2519	0.255	10.9685
15	LSKnet-lenet	0.0013	0.0283	1.4311
16	LSKnet-MLP	0.0026	0.02	1.0461
17	LSKnet-Resnet	0.143	0.3175	14.9091
18	EMA-Alex	0.257	0.3316	15.3044
19	EMA-lenet	0.0085	0.0832	4.2422
20	EMA-MLP	0.0058	0.0588	2.9846
21	EMA-VGG16	0.0265	0.139	7.1516
22	ReLu-Alexnet	0.2523	0.2917	13.0623
23	ReLu-MLP	0.0075	0.0198	1.1345
24	ReLu-Resnet	0.0405	0.1565	7.8879
25	CSFAM-MLP	0.0004	0.0141	0.7219

Note: ↓ indicates lower is better.

**Table 8:** The results of the ablation experiment.

NO.	Channel	Spatial	MSE ↓	MAE ↓	SMAPE ↓	R^2^ ↑	Standard Deviation
1			0.0089	0.0774	3.9733	0.518	0.722
2	✓		0.0023	0.0164	0.8638	0.8776	0.390
3		✓	0.0026	0.02	1.0461	0.8575	0.332
4	✓	✓	0.0004	0.0141	0.7219	0.9766	0.182

Note: ↑ indicates higher is better; ↓ indicates lower is better; ✓ denotes that the module is applied.

**Table 9:** Comparison of Convolutional Layers in the Adaptive Channel Fusion Module.

Channel Module Variant	Total Module Parameters	Relative Increase/Decrease vs. Multi-Scale	Test Set R^2^	Test Set MAE (Normalized)	Test Set MSE
Multi-scale (1 × 1 + 3 × 3 + 5 × 5)	815	—	0.9766	0.0141	0.0004
Only 1 × 1 Convolution	635	decrease of about 22%	0.9278	0.0245	0.0013
Only 3 × 3 Convolution	685	decrease of about 16%	0.9548	0.0159	0.0008
Only 5 × 5 Convolution	765	decrease of about 6%	0.9630	0.0173	0.0007

**Table 10:** Comparison of Neural Networks.

NO.	Model Name	MSE ↓	MAE ↓	SMAPE ↓
1	Alex	0.2905	0.3187	14.3698
2	Lenet	0.0109	0.0925	4.7089
3	MLP	0.0089	0.0774	3.9733
4	Resnet	0.1755	0.4021	18.1877
5	VGG	0.0338	0.1523	7.8172
6	Vit	0.0081	0.0716	3.6482
7	Dense	0.0332	0.1449	7.065
8	Mobilenet	3.549	1.8781	63.6219
9	CNN	0.0914	0.2718	12.6182
10	CSFAM-MLP	0.0004	0.0141	0.7219

Note: ↓ indicates lower is better.

## Data Availability

The data that support the findings of this study are available from the corresponding author upon reasonable request.

## Appendix A

In high-rise building fire wind-field experiments, the theoretical basis for the numerical setting of heat sources is Technical Standard for Building Smoke Control and Exhaust System, jointly issued by the Ministry of Housing and Urban-Rural Development of the People’s Republic of China and the General Administration of Quality Supervision, Inspection and Quarantine of the People’s Republic of China. The table below provides the reference parameters for the heat-source settings.

**Table A1:** Technical Standard for Smoke Management Systems in Buildings.

Building Category	Sprinkler Status	Heat Release Rate Q (MW)
Offices, classrooms, guest rooms, corridors	No sprinkler	6.0
	With sprinkler	1.5
Shops, exhibition halls	No sprinkler	10.0
	With sprinkler	3.0
Other public places	No sprinkler	8.0
	With sprinkler	2.5
Parking garages	No sprinkler	3.0
	With sprinkler	1.5
Industrial buildings	No sprinkler	8.0
	With sprinkler	2.5
Warehouses	No sprinkler	20.0
	With sprinkler	4.0
